# Deciphering the phosphorylation-based regulatory strategies of *Haemaphysalis longicornis* in heat stress

**DOI:** 10.1186/s13071-025-07025-1

**Published:** 2025-09-24

**Authors:** Xiao-Li Zhang, Ning-Mei Wang, Bo-Fang Zhang, Abolfazl Masoudi, Jia-Xuan Han, Ye-Fei Zhang, Tong Li, Chang-Ying Ding, Zi-Dan Wang, Jing-Ze Liu, Hui Wang

**Affiliations:** 1 Ministry of Education Key Laboratory of Molecular and Cellular Biology, Hebei Key Laboratory of Animal Physiology, Biochemistry and Molecular Biology, College of Life Sciences, Hebei Normal University, Shijiazhuang, 050024 Hebei Province China; 2 Department of Pathogenic Biology, College of Basic Medicine, Chengde Medical University, Chengde, 067000 Hebei Province China; 3 Department of Biological Sciences, University of Illinois, Chicago, IL USA; 4 Hebei Xiaowutai Mountain National Nature Reserve Management Center, Zhangjiakou, 075700 Hebei Province China

**Keywords:** *Haemaphysalis longicornis*, Adaptation, Phosphoproteomics, High temperature

## Abstract

**Background:**

The Asian hard tick (*Haemaphysalis longicornis*) is an obligate hematophagous ectoparasite belonging to the family Ixodidae (hard ticks). This species serves as a competent vector for numerous highly pathogenic agents. The number of ticks that survive the summer, particularly under high-temperature conditions, exerts immediate and lasting impacts on tick populations and tick-borne disease prevalence. Therefore, it is crucial to study how high temperatures affect ticks, as well as how ticks adopt effective behavioral strategies and physiological adaptations to cope with heat stress. Phosphorylation, a kind of important protein post-translational modification (PTM), is vital for cellular signal transduction, gene expression, and rapid cell cycle regulation.

**Methods:**

This study systematically analyzed phosphorylation changes in proteins from the salivary gland, midgut, ovary, and Malpighian tubules of ticks exposed to different temperatures (26 °C, 36 °C, and 45 °C) using quantitative proteomics. Differentially expressed phosphoproteins were comprehensively assessed using bioinformatics tools, supplemented with ribonucleic acid (RNA) interference and tick survival assays to validate key protein functions.

**Results:**

This study reveals a tissue-specific phosphorylation regulatory pattern. It identifies the involvement of kinase families such as CK1, AGC, and CMGC in the heat stress response. Phosphorylation modifications of spliceosome components and upregulated Hsp90 phosphorylation were found to regulate RNA splicing pathway and heat shock response, respectively. Notably, the Hsp90 co-chaperone CDC37 was critical for maintaining GRK stability and ensuring tick survival under high-temperature conditions.

**Conclusions:**

The thermal stress response in *H. longicornis* involves a coordinated network of protein kinases, alternative splicing events, and heat shock proteins along with their co-chaperones. These findings provide a foundation for further deciphering of the molecular regulatory mechanisms of tick tolerance to high temperatures.

**Graphical abstract:**

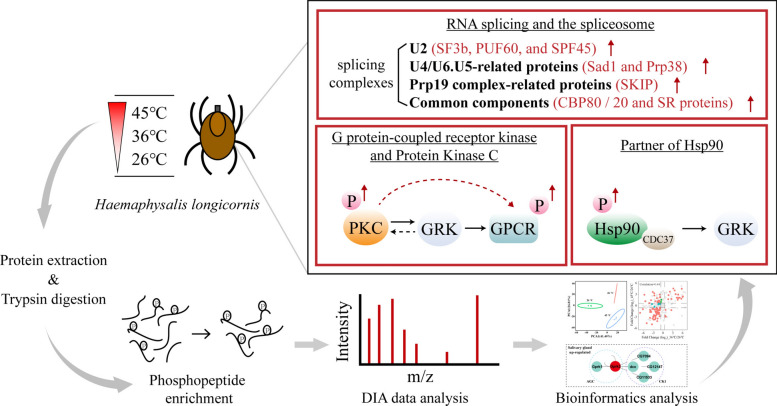

**Supplementary Information:**

The online version contains supplementary material available at 10.1186/s13071-025-07025-1.

## Background

Ticks are obligate ectoparasitic hematophages whose survival requires repeated blood meals from vertebrate hosts and that belong to the class Arachnida, subclass Acari, superorder Parasitiformes, and order Ixodida [1, 2]. They are globally distributed and can transmit a variety of highly pathogenic agents, posing significant threats to human and wildlife health, as well as livestock production [3, 4]. *Haemaphysalis longicornis* is primarily found in East Asia and Australia [5]. In recent years, several states in the USA have also reported the invasion of this species [6]. *H. longicornis* serves as a vector for multiple pathogens, including *Theileria*, *Babesia*, *Anaplasma*, *Rickettsia*, and so on [7–10].

Most of the tick life cycle occurs during the non-parasitic stage [11]. The external environment significantly influences the growth, development, and reproduction of ticks [12]. Temperature, as a critical environmental factor, directly affects tick molting, oviposition, and reproduction [13–15]. Through years of research, we have identified 26 °C as the optimal temperature for *H. longicornis* survival [16]; 36 °C was selected as it corresponds to the highest average summer temperature at the tick collection site and approximates host surface temperatures [17]. Our findings demonstrate that the upper thermal tolerance threshold of *H. longicornis* is 45 °C.

Summer, characterized by high temperatures and extreme weather events, plays a critical role in determining tick survival rates [18]. The number of ticks that endure summer conditions directly influences population dynamics and the transmission intensity of tick-borne diseases in both current and subsequent years [19]. Studies have shown that rising temperatures increase tick activity, leading to the expansion or shift of tick distribution ranges [20]. Additionally, higher temperatures increase host-seeking aggression in ticks, thereby elevating the incidence and broadening the endemic areas of tick-borne diseases [21]. With the continuous enhancement of the greenhouse effect, it is particularly important to study how high temperature affects ticks and how ticks adopt effective behavioral strategies and physiological adjustment mechanisms to cope with continuous high-temperature weather.

To survive and reproduce under high-temperature stress, ticks have evolved unique physiological regulation mechanisms [22, 23]. When exposed to high temperatures without the ability to escape, ticks, like other arthropods, rely on a thick chitinous exoskeleton to prevent rapid water loss [24]. Simultaneously, key tissues such as the salivary glands, midgut, ovaries, and Malpighian tubules actively engage in physiological and molecular regulatory strategies to maintain normal bodily functions and mitigate heat-induced damage [25]. For instance, the salivary glands regulate water balance by secreting saliva with high ionic concentrations, facilitating water absorption from the air [26]. The Malpighian tubules not only aid in rapid excretion but also help conserve water and reduce water loss during dehydration [27]. However, to our knowledge, there are limited studies on the molecular regulatory mechanisms of tick salivary glands, midgut, ovaries, and Malpighian tubules under high-temperature stress, particularly regarding protein regulation and post-translational modifications (PTMs).

PTMs of proteins are essential for regulating protein function [28]. The remarkable complexity and diversity of biological functions, orchestrated by just over 10,000 proteins in the body, are largely achieved through multiple PTMs [29]. Approximately 300 distinct PTM types are known to regulate physiological activities across the organism [30]. Among these, phosphorylation, the most common and significant form of PTMs, plays a vital role in enabling rapid physiological responses to environmental and cellular changes [31].

To systematically analyze the protein regulatory mechanisms of ticks under thermal stress, we performed data-independent acquisition (DIA) proteomics to analyze phosphorylation alterations in *H. longicornis* tissues. Salivary glands, midgut, ovary, and Malpighian tubules were examined after 5-h exposures to 26 °C, 36 °C, and 45 °C. This study holds significant importance for comprehensively understanding the effects of global warming and extreme high-temperature environments on ticks.

## Methods

### Feeding of ticks

A bisexual *H. longicornis* population was field-collected from the Xiaowutai Mountain National Nature Reserve (Zhangjiakou City, Hebei Province, China). During non-feeding periods, the ticks were maintained in a constant-temperature incubator (26 °C and 75% humidity). For blood feeding, 20 adult ticks were placed on each ear of New Zealand white rabbits. Rabbits’ ears were wrapped with white cloth to prevent tick escape. All procedures involving animals were performed following protocols approved by the Animal Ethics Committee of Hebei Normal University (Approval No. IACUC-177026).

### Tissue collection

**Fig. 1:**
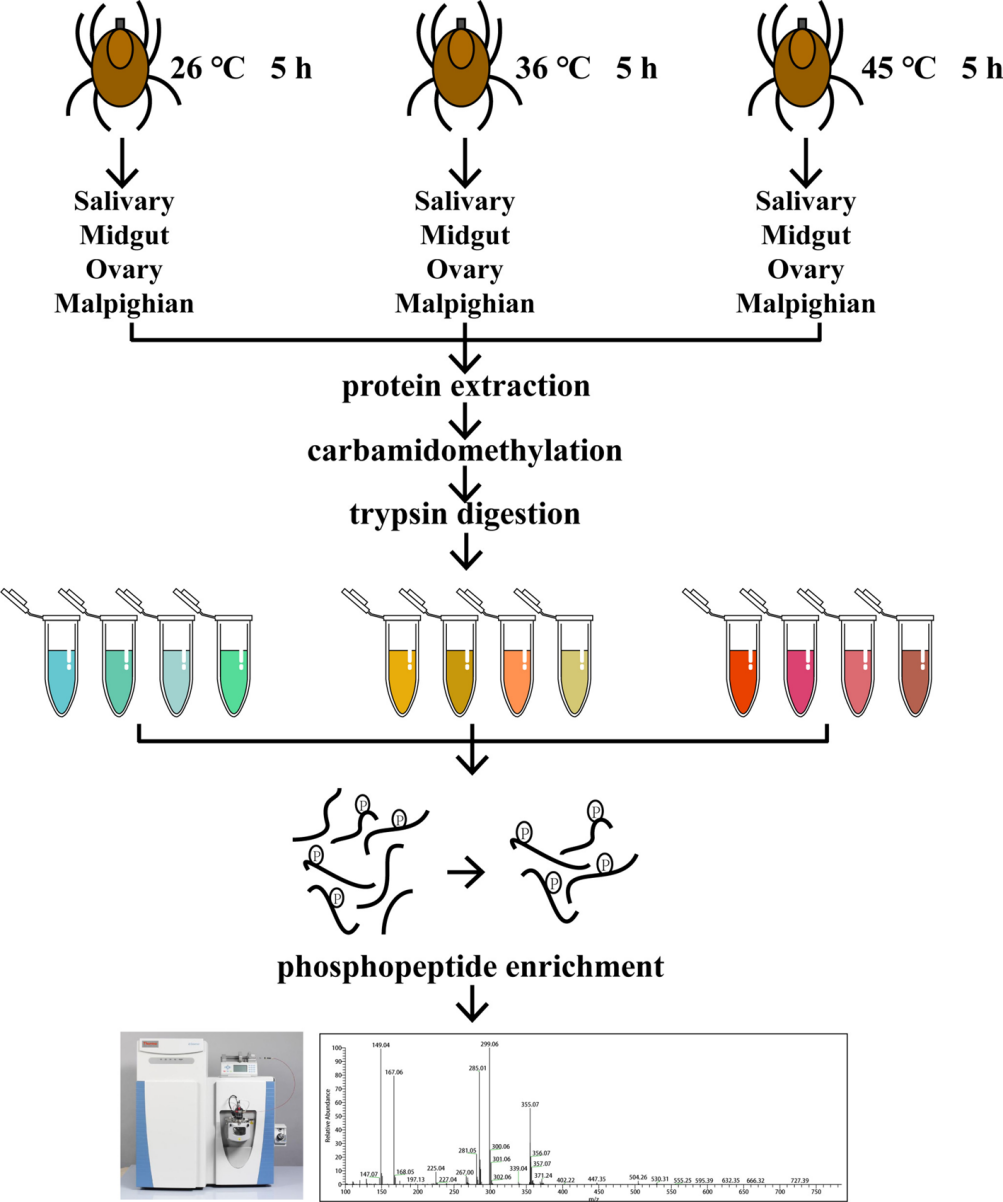
Overall experimental design and process

The experimental design is illustrated in Fig. 1. Partially fed ticks (2.5 days post attachment, measuring 0.48 ± 0.02 cm, and weighing 11.9 ± 0.4 mg) were removed from the rabbit ears and immediately transferred to incubators for 5 h. The incubator was programmed with temperature settings of 26 °C, 36 °C, and 45 °C, with a ramping rate of 0.5 °C per minute. Laboratory tests revealed that *H. longicornis* could survive at 45 °C for more than 5 h, but experienced mass mortality when the temperature was increased by 1 °C. At each temperature condition, 20 ticks were placed for treatment. Surface sterilization of ticks was performed by 1-min ethanol (70%) immersions [32]. The back of the tick was gently scratched with a scalpel, and its tissues were carefully dissected under a stereoscopic microscope (SZ61, Olympus, Japan) using fine tweezers (0208-5-PO, Dumont, Switzerland). Dissected tissues were placed in 0.01 M phosphate-buffered saline (PBS) containing 0.05 M NaF, 0.01 M Na_3_VO_4_, and 0.01 M protease inhibitor cocktail (Roche, Germany), then quickly stored at −80 °C.

### Protein extraction

Different tissues were thoroughly homogenized in a 1.5-mL tube using a grinding rod, respectively. The tissue homogenate was centrifuged for 10 min (4 °C, 12,000×*g*). The subsequent protein extraction protocol followed our previously described method [33]. Briefly, the supernatant was mixed with 3 mL Tris-saturated phenol (pH 7.8) in a new 15-mL centrifuge tube. After centrifugation (12,000×*g*, 10 min, 4 °C), the aqueous phase was carefully discarded. The phenolic phase was then washed with 3 mL 50 mM Tris–HCl (pH 8.0) by vigorous vortexing (1 min), followed by repeat centrifugation under identical conditions. Following phenol extraction, the aqueous phase was carefully removed and proteins were precipitated by adding 5 volumes of 0.1 M ammonium acetate in ice-cold methanol. After storage at −20 ℃ overnight, samples were centrifuged (12,000×*g*, 10 min, 4 °C). The protein pellet was washed twice with 1 mL ice-cold methanol. Finally, the purified protein was lyophilized and stored at −80 °C.

### Protein digestion

Each sample (1 mg) was treated with 10 mM dithiothreitol at 37 °C for 60 min. After reduction, alkylation was carried out with iodoacetamide (20 mM) in darkness at 26 °C for 30 min. Trypsin (Promega, USA) was used to digest the pretreated proteins at a ratio of 1:20 (enzyme:protein) at 37 °C for 12 h. The resulting peptides were desalted using C18 column (Anpel Laboratory Technologies, China) and vacuum-dried. Digestion efficiency was verified by nanoLC–MS/MS analysis (Q Exactive HF, Thermo Fisher Scientific, USA).

### Enrichment of phosphopeptides

Phosphopeptides were enriched using TiO_2_ (GL Sciences, Japan) that had been washed two times with an acetonitrile buffer (3% trifluoroacetic acid saturated with glutamic acid). Subsequently, 800 μL desalted peptides were incubated with the beads at a 4:1 (w/w) ratio for 1 h under gentle rotation at 25 °C. After binding, the beads underwent three stringent wash steps: first with 1 mL 50% acetonitrile (ACN) solution, then twice with 1 mL 50% ACN containing 20 mM ammonium acetate. Elution was carried out in three steps: first with 200 μL 0.3 M NH_4_OH, then with two separate 200 μL of 0.5 M NH_4_OH. After lyophilization to complete dryness, the samples were kept at −80 °C until liquid chromatography–tandem mass spectrometry (LC–MS/MS) analysis.

### DIA quantitative proteomic analysis

Four tissues of semi-engorged female ticks exposed to different temperatures (26 °C, 36 °C, and 45 °C) were quantitatively analyzed using DIA proteomics. Each experiment was performed in triplicate. Peptide samples were resuspended in loading solvent (99.9% water/0.1% formic acid) spiked with indexed retention time (iRT) reagent (Biognosys, Switzerland). Quantitative DIA analysis was conducted on a nanoLC-MS/MS platform consisting of a Waters M-Class ultra performance liquid chromatography (UPLC) (Waters, USA) interfaced with a Q Exactive high-field (HF) mass spectrometer (Thermo Fisher Scientific, USA). Peptide samples were first loaded onto a trap column (C18, 5 μm, 100 Å, 180 μm × 20 mm; Waters) and then analyzed on a column (C18, 1.8 μm, 75 μm × 250 mm; Waters). Separation was performed at a flow rate of 300 nL/min using a 120-min nonlinear gradient: 2–8% B (0–6 min), 8–35% B (6–120 min). Mobile phases consisted of: (a) 0.1% (v/v) formic acid in water and (b) 0.1% (v/v) formic acid in acetonitrile. Samples were electrosprayed into the Q Exactive HF. The Q Exactive HF was performed in DIA mode using the parameters: (a) MS1: m/z 350–1,200 (resolution: 60,000); (b) maximum ion injection time: 50 ms; (c) mass spectrometry stage 1 (MS1) automatic gain control (AGC) target: 3 × 10^6^; (d) DIA segmentation: one full MS1 scan (350–1,250 m/z) followed by 20 mass spectrometry stage 2 (MS2) scans; and (e) MS2 AGC target: 1 × 10^6^ (resolution: 30,000).

DIA raw data were processed using Spectronaut 15.0 (Biognosys, Switzerland) with the following analytical parameters: (a) false discovery rate (FDR) < 1% and (b) tryptic digestion allowing two or fewer missed cleavages. Searches were performed against a custom *H. longicornis* protein database (National Center for Biotechnology Information [NCBI] accession: GHLT00000000) supplemented with contaminant sequences from rabbit (*Oryctolagus cuniculus*) and human keratins. Post-translational modifications included variable modifications (phosphorylation [S/T/Y], *N*-terminal acetylation, and oxidation [M]) and fixed modification (carbamidomethylation [C]). Statistical significance thresholds were set at fold change > 1.5 and *p* < 0.05 for differential expression analysis.

### Bioinformatic analyses

We employed comprehensive bioinformatic approaches to analyze protein phosphorylation regulation in female tick tissues under thermal stress. Protein expression patterns across the four tissues were clustered using the “Mfuzz” R package [34]. The “pacman” R package was used to perform principal component analysis (PCA). Gene Ontology (GO) functional categorization was conducted using the Cloud Wu Kong website (https://www.omicsolution.org), which is based on the *Ixodes scapularis* database. The Kyoto Encyclopedia of Genes and Genomes (KEGG) database (https://www.kegg.jp/kegg/) was used to identify pathways. Phosphopeptides with precisely identified phosphorylation sites were submitted to the Cloud Wu Kong website to identify enriched phosphorylation motifs [35]. Site-specific kinase–substrate interactions were predicted using the iGPS software package [36]. Network diagrams were generated using Cytoscape software [37].

### RNA interference

The gene sequences selected for RNA interference (RNAi) were obtained from the *H. longicornis* whole genome database (GeneBank accession no.: GCA_013339765.2) using local BLAST. The GenBank accession nos. for G protein-coupled receptor kinase (*GRK*), protein kinase C (*PKC*), and cell division cycle 37 (*CDC37*) are KAH9362860.1, KAH9383540.1, and KAH9380665.1, respectively. Specific messenger RNA (mRNA) regions (*GRK*: 829–1,216 bp; *PKC*: 977–1,319 bp; and *CDC37*: 131–586 bp) were used for double-strand RNA (dsRNA) synthesis. Table 1 presents the primers used to clone target mRNA and generate dsRNA. For dsRNA synthesis, we followed the T7 RiboMAX^™^ Express RNAi System (Promega, USA) protocol. The dsRNA was validated by injection, as described in previously published studies [16]. Briefly, surface-sterilized ticks were injected with 2 μL dsRNA solution (1 μg/tick) into the hemocoel of unfed females using a 10-μL Hamilton microliter syringe (Reno, USA). Control ticks were injected with green fluorescent protein (GFP; GenBank accession no.: KX247384) dsRNA. At 24 h post injection, total RNA was extracted from four tissues, followed by complementary DNA (cDNA) synthesis. RNAi efficiency was assessed using quantitative reverse transcription polymerase chain reaction (qRT-PCR).

**Table 1:** The primers for cloning target mRNA and synthesizing dsRNA

Primer	Sequence (5′–3′)
CDC37-F	AGAGGACGAGGTAGTTGGACGATGAAGATGAGACGC
CDC37-R	AGAGGACGAGGTAGTTGGAGAGGACGAGGTAGTTGG
GRK-F	TAATACGACTCACTATAGGGAGGAAAATCATTACGCAA
GRK-R	TAATACGACTCACTATAGGGCCTCTCCCAGGACACACG
PKC-F	AGAGGACGAGGTAGTTGGCCAAGCACCCTTTCCTCA
PKC-R	AGAGGACGAGGTAGTTGGATCTCGGGCGCAATGTAG

T7 promoters are marked by single underlining.

### qRT-PCR analysis after RNAi

The interaction among *GRK*, *PKC*, and *CDC37* was examined through qRT-PCR analysis. Each sample was derived from at least three ticks, and three independent replicates were performed. The qRT-PCR primers are presented in Table 2. β-actin (GenBank accession no.: AY254898), which maintains stable expression under high-temperature conditions, was employed as an internal reference. The cDNA synthesized in the previous step was used as the template. qRT-PCR (CFX96 System, Bio-Rad USA) was executed with SYBR Master Mix (Vazyme, China) under standard reagent conditions. The resulting data were quantified using the 2^−ΔΔCt^ method [38].

**Table 2:** The fluorescent quantitative primers used for qRT-PCR in this study

Primer	Sequence (5′–3′)
CDC37-qF	ATGGACGACAAACACGAC
CDC37-qR	ACTGCTGGTCCGCAATCT
GRK-qF	GAGCGATAAGACGAGGCA
GRK-qR	GGAGCAGTGGGTGGAGTT
PKC-qF	TCACCTCCCTACAACCCT
PKC-qR	CAGCCCTCCACTGTCTTT
β-actin-qF	CGTTCCTGGGTATGGAATCG
β-actin-qR	TCCACGTCGCACTTCATGAT

### Tick survival rate analysis after RNAi

A survival rate experiment was conducted to evaluate the effects of *CDC37* gene interference on the survival of *H. longicornis* under high-temperature conditions. Four groups were established, each consisting of 20 randomly selected ticks. Gene interference for *CDC37* was performed using the methods described previously, while interference of the *GFP* gene served as the control group. For each group, dead ticks were counted twice daily at 12-h intervals, and survival rates were calculated over a 7-day period. Ticks were classified as dead when they showed no coordinated appendage movement following CO_2_ stimulation.

### Statistical analyses

Statistical analyses were performed using Student’s *t*-tests for most comparisons, with significance thresholds set at *P* < 0.05 (^*^P < 0.05, ^**^P < 0.01). Survival data were analyzed by log-rank (Mantel–Cox) test. All experiments included at least three biological replicates.

## Results and discussion

### Data quality control and quantitative analysis of phosphorylated proteins

**Fig. 2:**
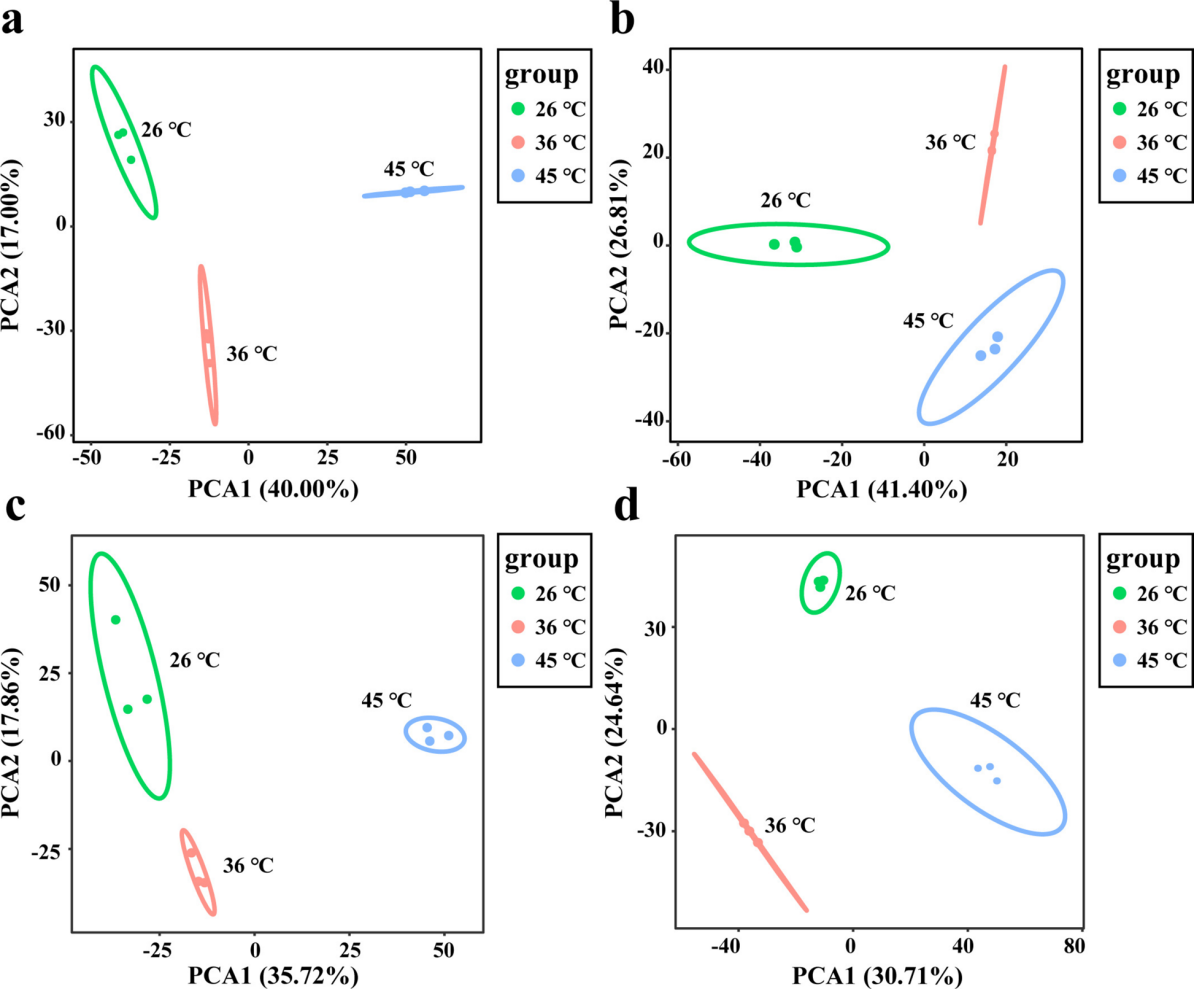
PCA analysis of phosphopeptides in salivary glands (**a**), midgut (**b**), ovary (**c**), and Malpighian tubules (**d**) under temperature stress at 26 °C, 36 °C, and 45 °C. Green indicates 26 °C treatment for 5 h, red indicates 36 °C treatment for 5 h, and blue indicates 45 °C treatment for 5 h

Phosphorylation sites in female tick tissues (salivary glands, midgut, ovary, and Malpighian tubules) were analyzed under different temperatures using bioinformatics. PCA revealed high reproducibility among replicates and significant differences between temperature groups (Fig. 2 and Additional File 6: Supplementary Table S1). These findings reflect the role of phosphorylation modifications in rapidly regulating physiological responses to high-temperature stress. Mass spectrometry data are available in ProteomeXchange (ID: PXD053687).

The box plot confirmed consistent normalized abundance of phosphorylated peptides across biological replicates (Additional File 1: Fig. S1). Phosphoproteomic analysis of *H. longicornis* revealed distinct tissue-specific patterns. The salivary glands contained 4203 phosphopeptides (1168 proteins) with 5501 phosphorylation sites, predominantly serine (89.06%). The midgut showed 1491 phosphopeptides (738 proteins), mostly monophosphorylated (93.02%). Ovaries (3756 phosphopeptides) and Malpighian tubules (4456 phosphopeptides) exhibited similar profiles. Across all tissues, monophosphorylation was most common (> 72%), followed by diphosphorylation, with minimal triphosphorylation (< 3.2%). (Additional File 2: Supplementary Fig. S2).

### Analysis of differential phosphorylated peptides

**Fig. 3:**
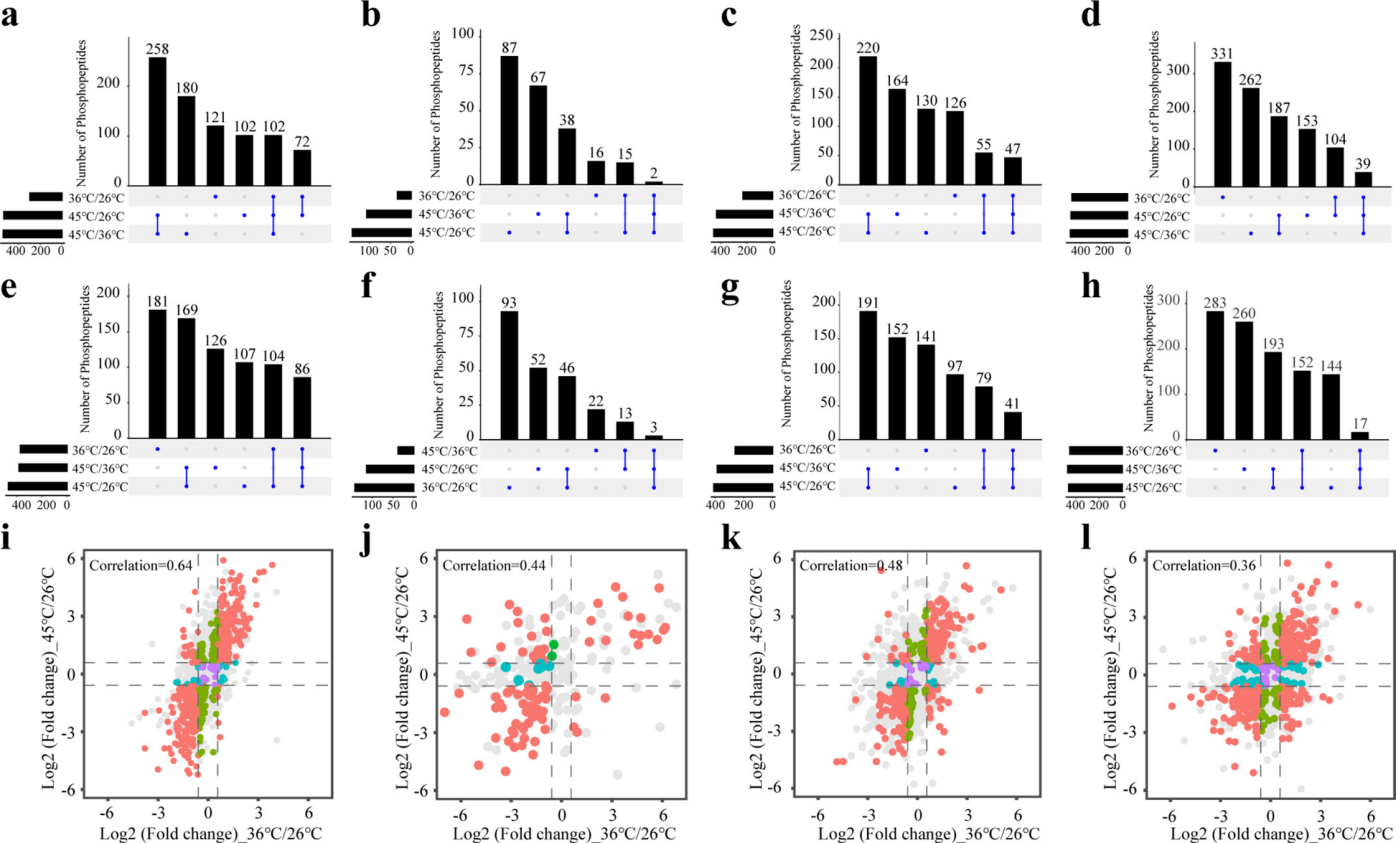
Intersection analysis of upregulated (**a–d**) and downregulated (**e–h**) phosphopeptides and correlation analysis (**i–l**) of phosphopeptides in salivary glands, midgut, ovary, and Malpighian tubules, respectively. Red indicates that the fold change of phosphopeptides in two groups was > 1.5 times; blue and green indicate that the fold change of phosphopeptides in one group was > 1.5 times, while another group was < 1.5 times; purple indicates that the fold change of phosphopeptides in two groups was < 1.5 times; and gray indicates phosphopeptides with *P*> 0.05

Temperature shifts induced distinct phosphopeptide expression changes across tick tissues. In salivary glands, 102 phosphopeptides were upregulated and 86 downregulated (36 °C/26 °C, 45 °C/26 °C, and 45 °C/36 °C), with a strong thermal response (correlation: 0.64). The midgut showed minimal changes (two upregulated, three downregulated; correlation: 0.44). Ovaries and Malpighian tubules displayed moderate regulation (ovaries: 47 upregulated, 41 downregulated, correlation: 0.48; Malpighian tubules: 39 upregulated, 17 downregulated, correlation: 0.36) (Fig. 3 and Additional File 7: Supplementary Table S2).

Cluster analysis of phosphorylated peptides in tick salivary glands, midgut, ovary, and Malpighian tubules across three temperatures revealed six distinct expression patterns, highlighting tissue-specific phosphorylation responses to thermal variation. As temperatures increased, the salivary glands exhibited 350 continuously upregulated and 317 downregulated peptides, while the midgut showed 47 up- and 50 downregulated peptides. Similarly, the ovary displayed 372 up- and 342 downregulated peptides, and the Malpighian tubules had 256 up- and 391 downregulated peptides (Additional File 3: Supplementary Fig. S3 and Additional File 8: Supplementary Table S3).

### Kinase and heat stress of *H. longicornis*

**Fig. 4:**
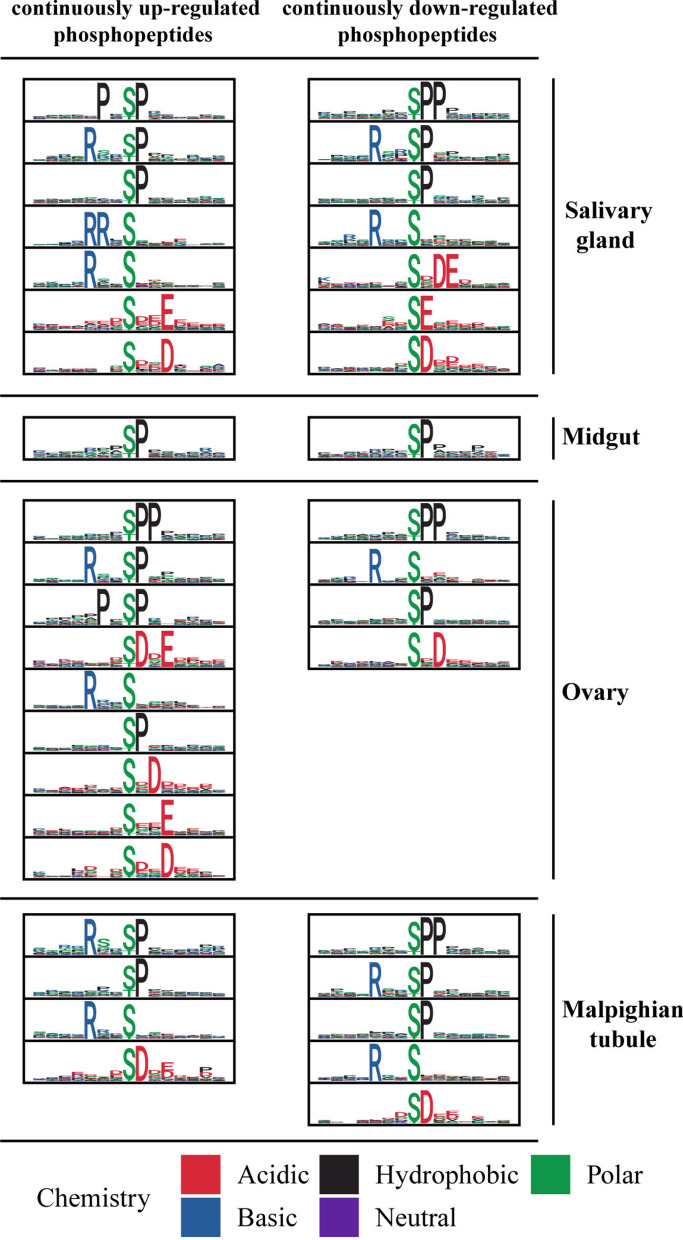
Motif analysis of differentially expressed phosphopeptides in salivary glands, midgut, ovary, and Malpighian tubules

Kinase substrate specificity is typically determined by specific amino acid sequence characteristics (motifs) surrounding the phosphorylation site. Different phosphorylation motifs often represent catalytic sites of distinct kinases [39]. Identifying kinase-specific phosphorylation sites is essential for elucidating phosphorylation-mediated regulatory mechanisms [40]. In *H. longicornis*, phosphorylation motif analysis revealed the conserved [-pS-P-] motif in all tissues. Additionally, salivary glands uniquely contained [-R-pS-P-] and [-R-pS-] motifs, whereas ovaries and Malpighian tubules shared [-R-pS-] and [-pS-D-] motifs. Notably, ovaries were distinguished by [-pS-PP-], while Malpighian tubules exclusively displayed [-R-pS-P-] (Fig. 4 and Additional File 9: Supplementary Table S4). These tissue-specific motifs imply distinct kinase targeting mechanisms underlying thermal responses.

**Fig. 5:**
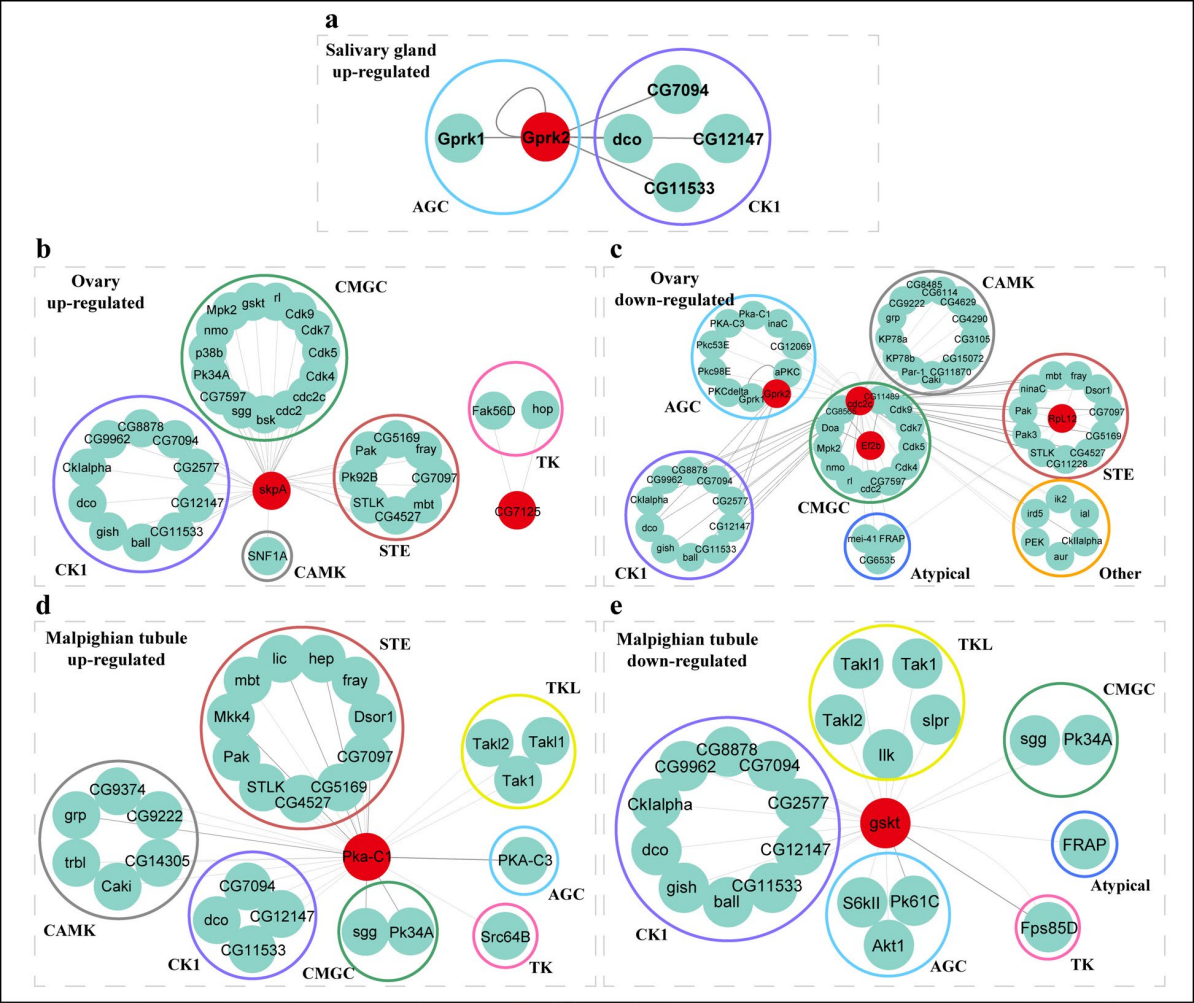
Kinase prediction of differentially expressed phosphorylated proteins in salivary glands (**a**), ovary (**b, c**), and Malpighian tubules (**d, e**). The red background indicates the phosphorylated protein identified in the experiment, the green background indicates the predicted protein kinase, and the circle indicates the kinase family

Kinase prediction analysis revealed CK1 as a conserved kinase family across all tick tissues under thermal stress. Salivary glands showed CK1 and AGC kinases for upregulated proteins, while ovaries exhibited CK1, CMGC, and STE families for upregulated proteins. Malpighian tubules involved CK1, CMGC, CAMK, and STE for upregulation (Fig. 5 and Additional File 10: Supplementary Table S5). No kinase predictions were obtained for midgut phosphoproteins.

**Fig. 6:**
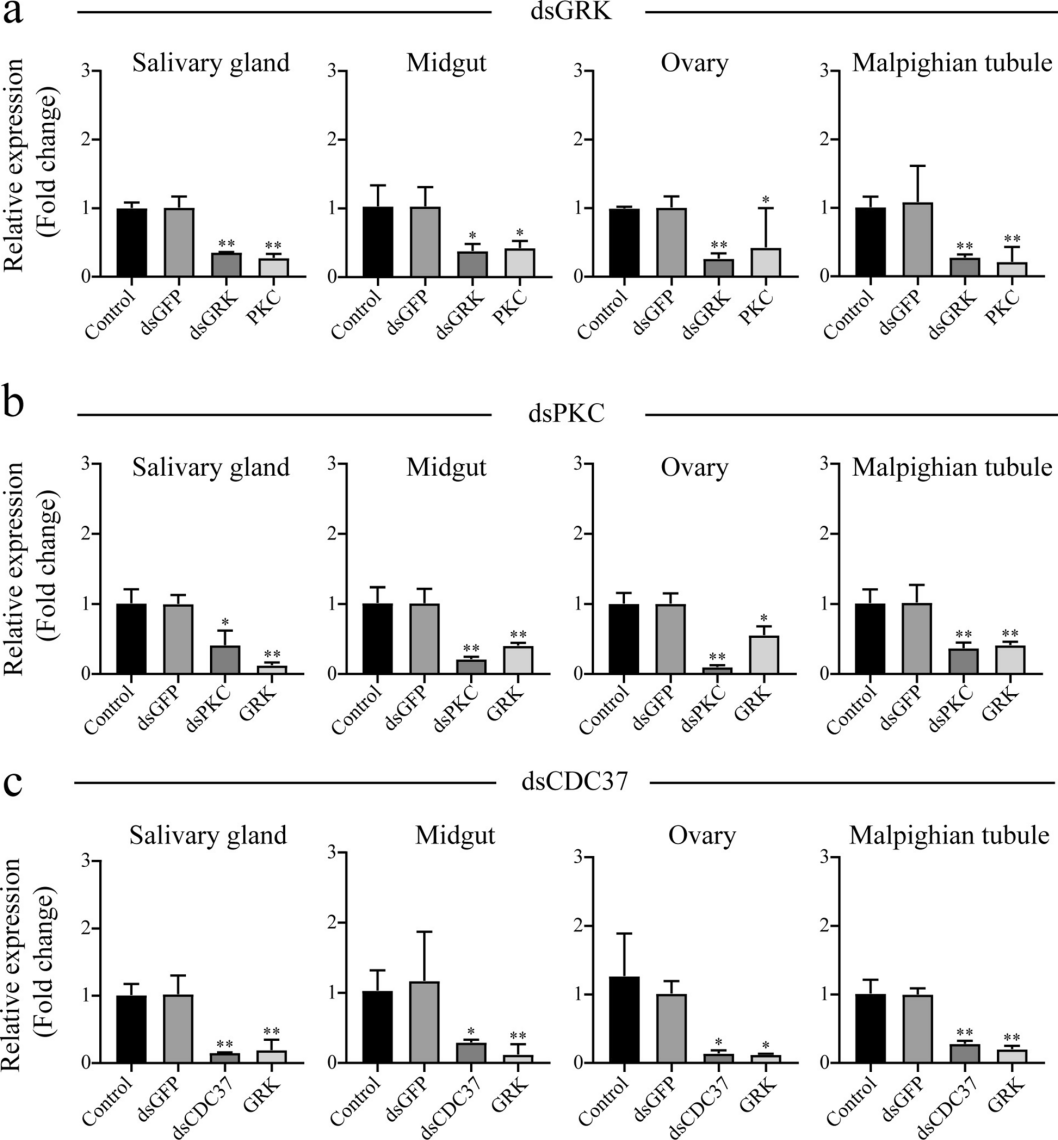
RNA interference of GRK (**a**), PKC (**b**), and CDC37 (**c**). The results are shown as means ± standard deviation (SD). Three independent repeats were performed. Compared with the green fluorescent protein (GFP) group. ^*^Statistically significant difference at *P* < 0.05;^**^Statistically significant difference at *P* < 0.01

Our study demonstrates that thermal stress induces significant phosphorylation of multiple protein kinases in ticks, particularly GRK and PKC, both members of the AGC kinase family. The activated GRK catalyzes the transfer of phosphate groups from ATP to specific serine/threonine residues, with notable specificity for intracellular domains of G protein-coupled receptor (GPCR) [41, 42]. After high-temperature stress, the phosphorylation of GPCR in various tissues of ticks changed. We speculate that this may be owing to a phenomenon in which ticks reduce the pressure on cells and the body to avoid excessive intracellular signal transduction. Previous study has demonstrated that PKC can phosphorylate and activate GRK, thereby mediating the desensitization of GPCR in turn [43]. Our RNAi experiments revealed that PKC expression was inhibited after interfering with GRK. After interfering with PKC, the expression of GRK was also inhibited (Fig. 6). This indicates that, on the one hand, the expression of GRK is regulated by PKC; on the other hand, GRK can also regulate the expression of PKC in turn.

Current literature lacks direct evidence linking GRK and PKC signaling pathways to arthropod thermotolerance. After interference with GRK and PKC, we analyzed the survival rate of *H. longicornis* but did not find a significant change before and after interference. We speculate that this may be because in addition to the GRK and PKC pathways, there are other pathways to regulate the heat tolerance of ticks and play the same function. In addition, the interference of GRK and PKC is not completely eliminated, and the residual GRK and PKC may also play a full role. The specific regulatory mechanism remains to be further verified by experiments.

We also predicted some kinases corresponding to continuously downregulated phosphorylated proteins. Ovaries exhibited calcium/calmodulin-dependent protein kinase (CAMK)/tyrosine kinase-like (TKL) families for downregulated proteins. Malpighian tubules involved CK1, TKL, and AGC for downregulation (Fig. 5 and Additional File 10: Supplementary Table S5). The underlying mechanisms may involve the following aspects: First, high temperature may disrupt the structural stability of kinases, particularly in the catalytic domains, leading to loss of enzymatic activity. For instance, the catalytic subunits of CK1 and AGC kinases are prone to aggregation or degradation under thermal stress [44, 45]. Second, elevated temperatures may suppress kinase gene transcription or impair mRNA translation, thereby reducing kinase protein expression. As observed in *Caenorhabditis elegans*, heat stress alters protein phosphorylation patterns involving kinase families such as CK1 and CAMK [46]. Third, thermal stress may induce protein denaturation, which could indirectly impair the activity of CK1, AGC, and other kinase families, ultimately leading to downregulation of protein phosphorylation [47]. We acknowledge that these potential mechanisms remain to be experimentally validated in our current study.

### Alternative splicing and heat stress of *H. longicornis*

GO and KEGG analysis showed that with increasing temperature, some phosphorylated proteins were mostly enriched in spliceosomes, regulating RNA splicing-related terms and pathways (Additional File 4: Supplementary Fig. S4, Additional File 5: Supplementary Fig. S5, Additional File 11: Supplementary Table S6, and Additional File 12: Supplementary Table S7). They included key components of the U2 complex (SF3b, PUF60, and SPF45), U4/U6.U5-related proteins (Sad1 and Prp38), Prp19 complex-related proteins (SKIP), and common components (CBP80/20 and serine and arginine-rich [SR] proteins). The main splicing complexes of eukaryotes contain five ribonucleoproteins, U1, U2, U4, U5, and U6, and a large number of auxiliary splicing factors [48]. Among these, serine and arginine-rich (SR) proteins, critical mediators of alternative splicing [49], exhibited significant phosphorylation changes across all four tick tissues under heat stress. Studies have shown that eukaryotes may interact directly with universal SR proteins through unique SR protein factors and fine-tune the processing and expression of important genes to cope with complex environmental changes [50, 51].

While no direct evidence links heat-induced alternative splicing to ticks, studies in other species demonstrate that thermal stress robustly influences splicing patterns. In rats, a 20.18% increase in alternative splicing events was observed under heat stress [52]. In heat-intolerant fish, thermal stress triggered a 29.2% rise in alternative splicing events and a 25.8% increase in alternatively spliced genes [53]. In *D. melanogaster*, alternative splicing of pre-mRNA is associated with high-temperature stress. Interrupting the processing of introns in alternative splicing can lead to death and developmental abnormalities in *Drosophila* [54]. Pre-mRNA splicing inhibition represents a conserved regulatory strategy deployed by diverse animal species to combat thermal stress [55, 56]. Importantly, this splicing inhibition does not represent passive thermal damage but rather constitutes an active survival strategy employed by organisms [57]. We speculate that the changes of phosphorylated proteins related to splicing and regulation of RNA splicing are mainly related to the resistance of ticks to high-temperature stress.

### Heat shock proteins and heat stress of *H. longicornis*

**Fig. 7:**
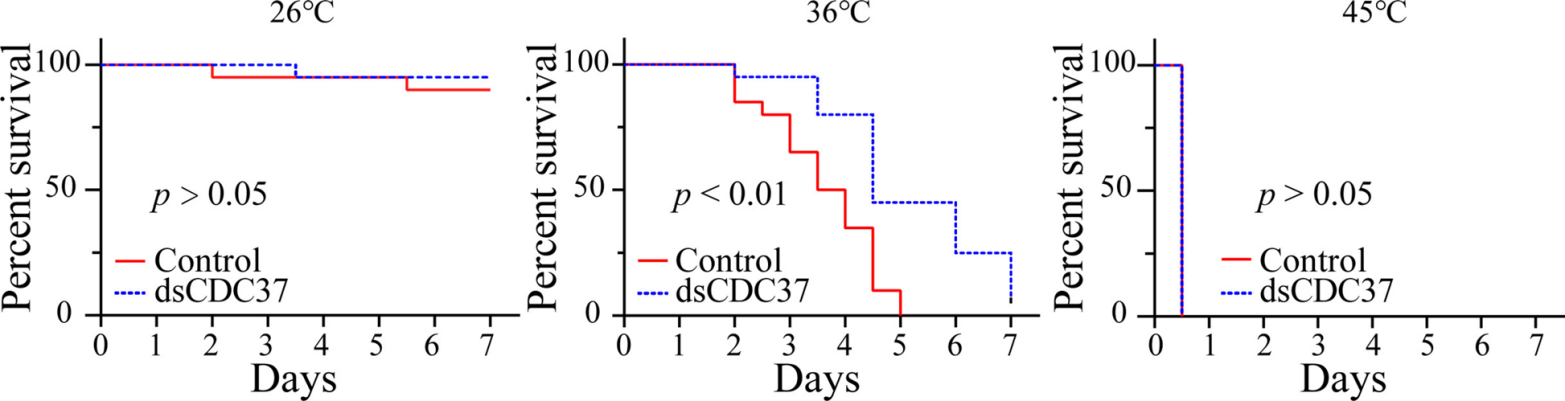
CDC37 improves *H. longicornis* survival rate. Three independent replicates with 20 ticks per sample

Heat shock proteins (Hsps) serve as crucial molecular components in the arthropod heat stress response system [58]. Among these, Hsp90 and Hsp70 exhibited significant upregulation under heat stress to protect cells from high temperature damage [59, 60]. For instance, in *Galleria mellonella* larvae, heat stress regulates extracellular signal-regulated kinase (ERK) and mitogen-activated protein kinase (MAPK) activity by enhancing Hsp90 phosphorylation levels [61]. In this study, we detected that the phosphorylation level of Hsp90 was significantly increased at high temperatures (salivary gland and ovarian). Literature review revealed that CDC37 is an important partner of Hsp90 and plays an important role in regulating protein kinases [62]. It can stabilize GRK and participate in the maturation of newly synthesized GRK protein, thus limiting the degradation of GRK protein [62]. It can also regulate the localization and transport of GRK in cells [63, 64]. Our RNA interference experiments demonstrated that CDC37 knockdown led to a marked reduction in GRK expression (Fig. 6). Furthermore, the survival rate showed that CDC37 could increase *H. longicornis* survival rates at high temperatures (Fig. 7). In the study of *Trypanosoma brucei*, it was found that the deletion of CDC37 may affect the function of Hsp90 [65]. While direct evidence linking CDC37 to arthropod heat stress is lacking, its crucial function within the Hsp90 pathway implies a potential significant role in arthropod stress responses.

## Conclusions

In this study, we systematically analyzed the phosphorylation changes of *H. longicornis* in response to high-temperature stress using quantitative proteomics. Using multilevel bioinformatic analysis methods, we elucidated the functions of protein kinases, alternative splicing, heat shock proteins, and their co-chaperones in the heat stress response of *H. longicornis*. These findings provide a basis for a comprehensive understanding of the molecular strategies employed by *H. longicornis* to adapt to global warming. They also offer potential molecular targets for anti-tick drugs in terms of controlling tick populations, thereby reducing the risk of tick-borne diseases.

## Supplementary Information

Additional file1. Figure S1 Abundance statistics between samples at 26 °C, 36 °C, and 45 °C in salivary glands (**a**), midgut (**b**), ovary (**c**), and Malpighian tubules (**d**). The abundance of phosphorylated peptides in the samples was log10 converted. The rhombus represents the average value.

Additional file2. Figure S2 The number of phosphopeptides identified with 1, 2, 3, 4, or 5 phosphorylation sites in salivary glands (**a**), midgut (**b**), ovary (**c**), and Malpighian tubules (**d**), respectively. The percentages of phosphorylation sites were serines, threonines, and tyrosines in salivary glands (**e**), midgut (**f**), ovary (**g**), and Malpighian tubules (**h**), respectively.

Additional file3. Figure S3 Cluster analysis of phosphopeptides in salivary glands (**a**), midgut **(b)**, ovary **(c)**, and Malpighian tubules (**d**) at 26 °C, 36 °C and 45 °C. Upper panel: each green line represents a phosphopeptide; black lines represent the overall trend of each cluster. Lower panel: each row represents a phosphopeptide, and each column represents an experimental treatment. Colors from green to red represent the phosphopeptide expression abundance from poor to rich, respectively.

Additional file4. Figure S4 GO annotation analysis of up-regulated (**a-d**) and down-regulated (**e-h**) phosphorylated proteins in salivary glands, midgut, ovary, and Malpighian tubules. MF denotes molecular function, BP denotes biological process, and CC denotes cell component.

Additional file5. Figure S5 KEGG pathway analysis of differentially expressed phosphorylated proteins in salivary glands (**a**), midgut (**b**), ovary (**c**), and Malpighian tubules (**d**).

Additional file6. Table S1 Quantitative search results of phosphorylated peptides.

Additional file7. Table S2 The number of differentially expressed phosphopeptides.

Additional file8. Table S3 Cluster analysis of phosphorylated peptides.

Additional file9. Table S4 The results of enriched phosphorylation motifs.

Additional file10. Table S5 The results of kinase prediction.

Additional file11. Table S6 The results of GO annotation of upregulated and downregulated phosphoproteins in four tissues.

Additional file12. Table S7 KEGG enrichment analysis of phosphoproteins in four tissues.

## Data Availability

The mass spectrometry proteomics data have been deposited to the ProteomeXchange Consortium (http://proteomecentral.proteomexchange.org) via the iProX partner repository [66] with the dataset identifier PXD053687.

## Abbreviations

PTMs: Post-translational modifications
DIA: Data-independent acquisition
ACN: Acetonitrile
AGC: Automatic gain control
FDR: False discovery rate
PCA: Principal component analysis
GO: Gene ontology
KEGG: Kyoto Encyclopedia of Genes and Genomes
RNAi: Ribonucleic acid interference
GRK: G protein-coupled receptor kinase
PKC: Protein kinase C
CDC37: Cell division cycle 37
dsRNA: Double-strand ribonucleic acid
GFP: Green fluorescent protein
SR: Serine and arginine-rich
GPCR: G protein-coupled receptor
Hsps: Heat shock proteins
