# Supplementary figures and images for: Deciphering the phosphorylation-based regulatory strategies of Haemaphysalis longicornis in heat stress
Source: Parasit Vectors. 2025 Sep 24;18:390. doi: 10.1186/s13071-025-07025-1 (PMC12461959; doi:10.1186/s13071-025-07025-1)

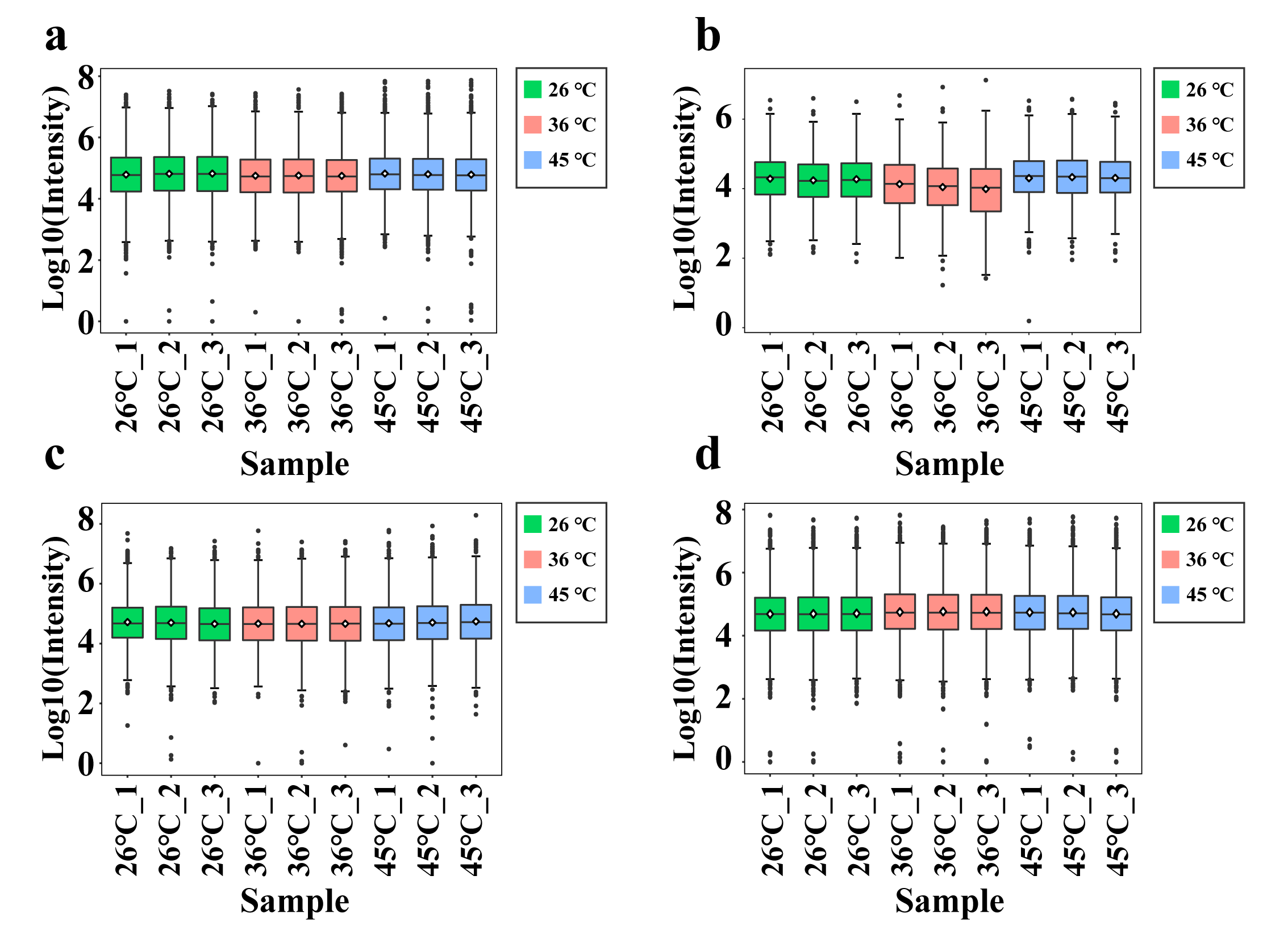

Supplement: Supplementary file 1 — Additional file1. Figure S1 Abundance statistics between samples at 26 °C, 36 °C, and 45 °C in salivary glands (a), midgut (b), ovary (c), and Malpighian tubules (d). The abundance of phosphorylated peptides in the samples was log10 converted. The rhombus represents the average value. [file 13071_2025_7025_MOESM1_ESM.tif]

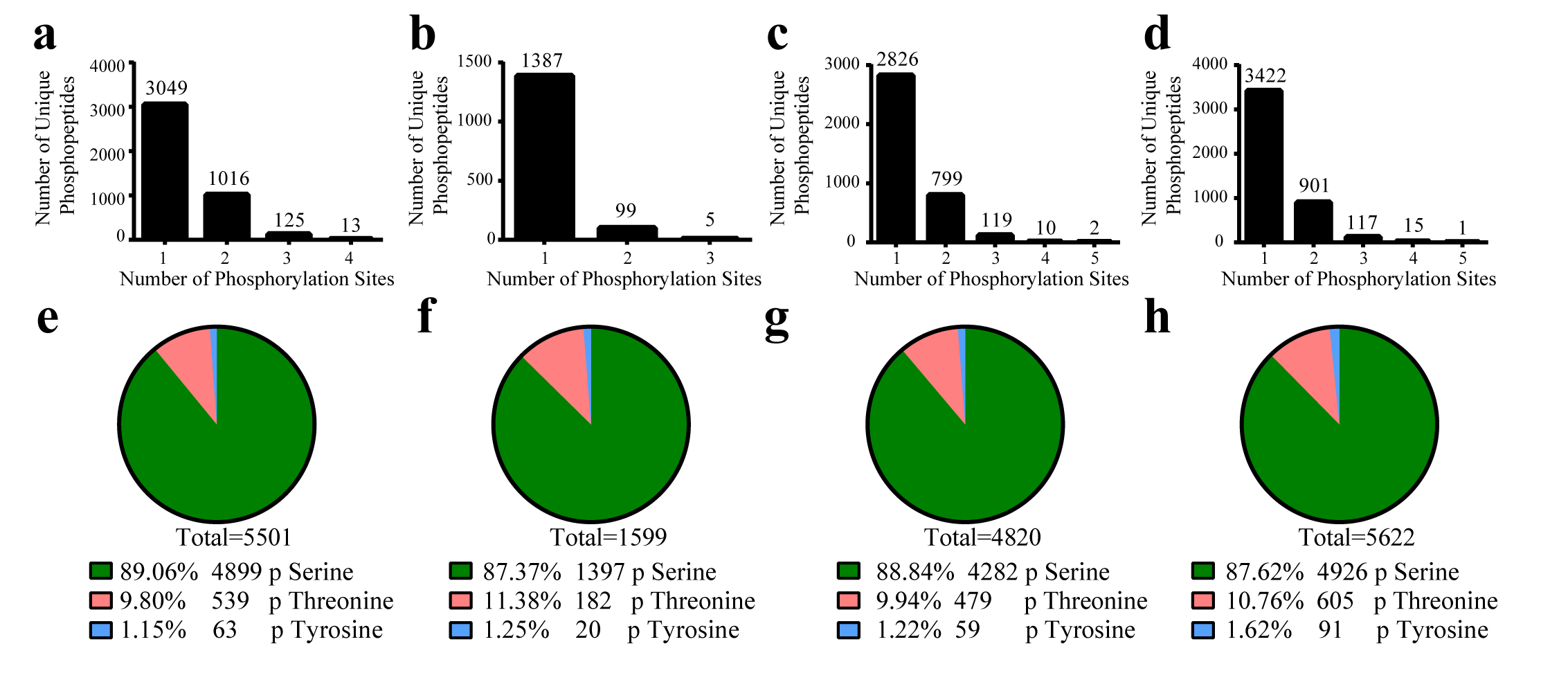

Supplement: Supplementary file 2 — Additional file2. Figure S2 The number of phosphopeptides identified with 1, 2, 3, 4, or 5 phosphorylation sites in salivary glands (a), midgut (b), ovary (c), and Malpighian tubules (d), respectively. The percentages of phosphorylation sites were serines, threonines, and tyrosines in salivary glands (e), midgut (f), ovary (g), and Malpighian tubules (h), respectively. [file 13071_2025_7025_MOESM2_ESM.tif]

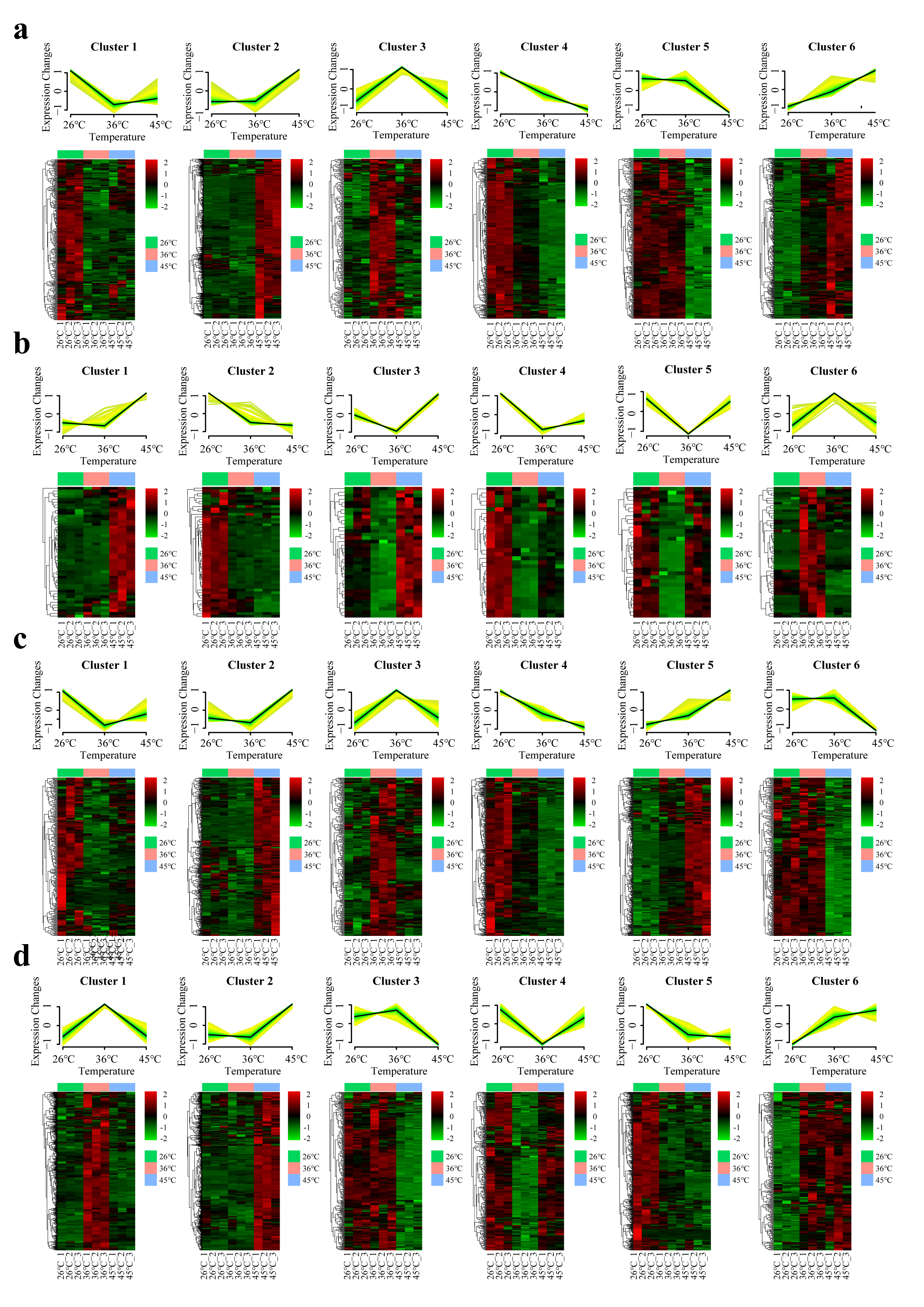

Supplement: Supplementary file 3 — Additional file3. Figure S3 Cluster analysis of phosphopeptides in salivary glands (a), midgut (b), ovary (c), and Malpighian tubules (d) at 26 °C, 36 °C and 45 °C. Upper panel: each green line represents a phosphopeptide; black lines represent the overall trend of each cluster. Lower panel: each row represents a phosphopeptide, and each column represents an experimental treatment. Colors from green to red represent the phosphopeptide expression abundance from poor to rich, respectively. [file 13071_2025_7025_MOESM3_ESM.tif]

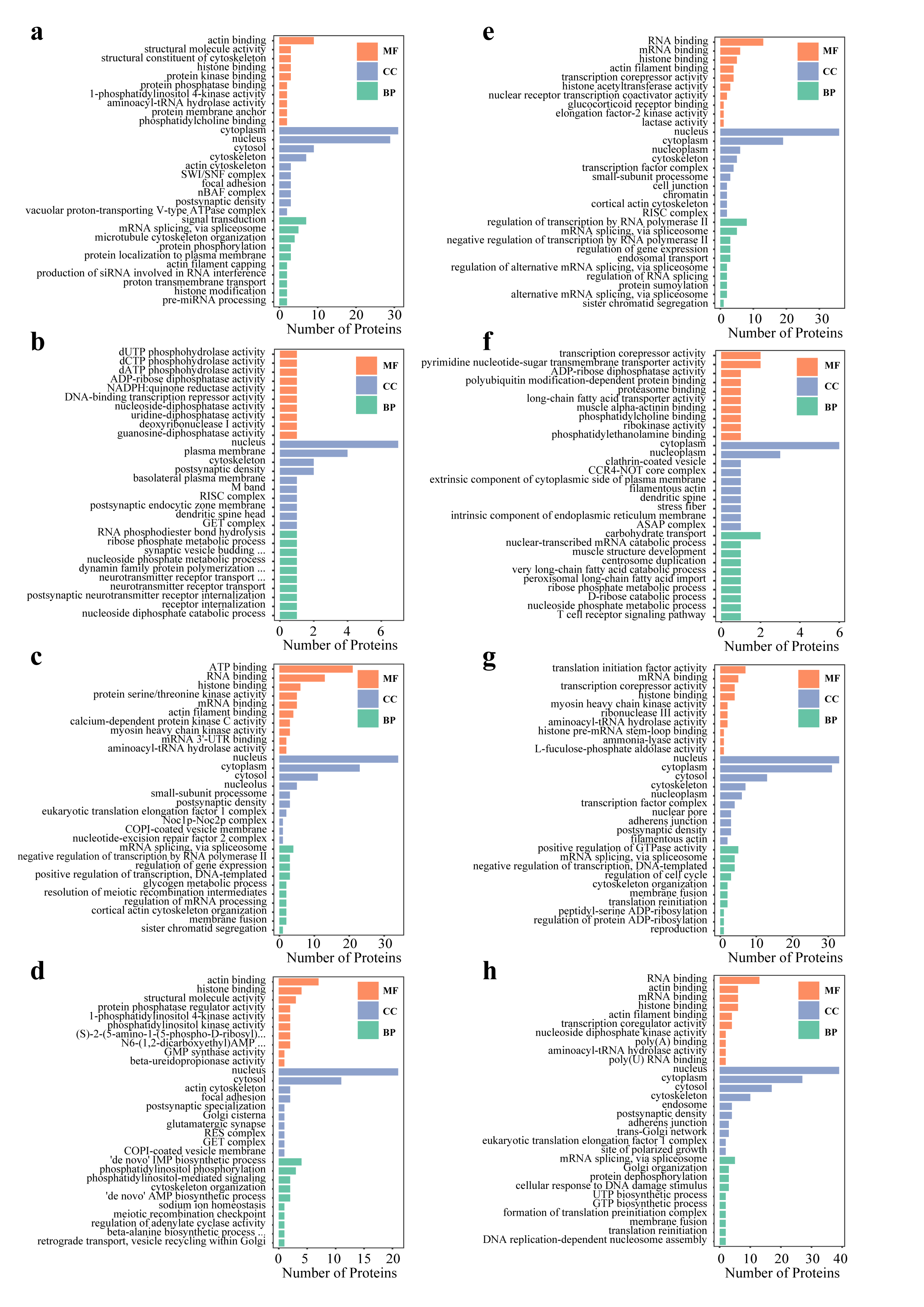

Supplement: Supplementary file 4 — Additional file4. Figure S4 GO annotation analysis of up-regulated (a-d) and down-regulated (e-h) phosphorylated proteins in salivary glands, midgut, ovary, and Malpighian tubules. MF denotes molecular function, BP denotes biological process, and CC denotes cell component. [file 13071_2025_7025_MOESM4_ESM.tif]

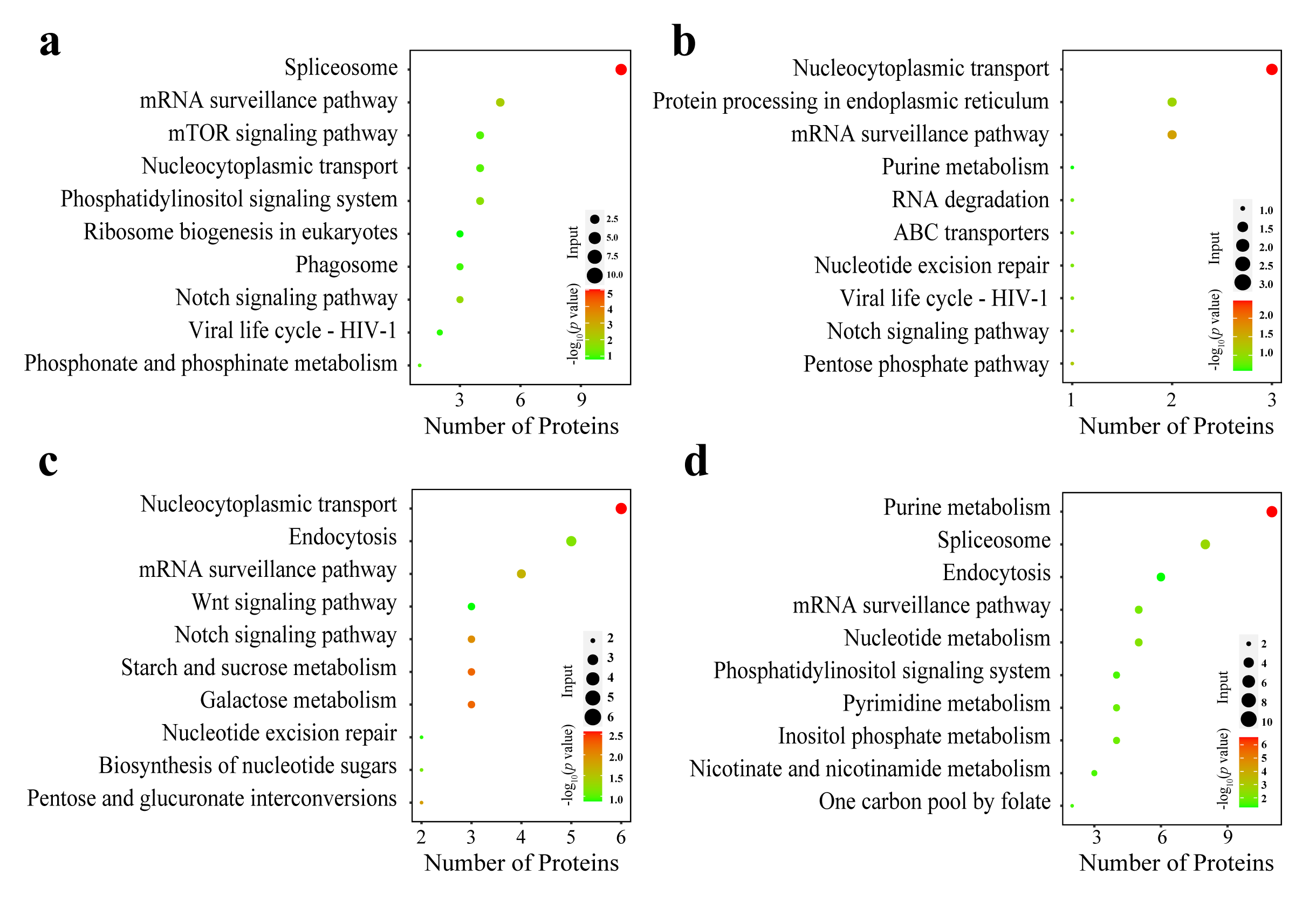

Supplement: Supplementary file 5 — Additional file5. Figure S5 KEGG pathway analysis of differentially expressed phosphorylated proteins in salivary glands (a), midgut (b), ovary (c), and Malpighian tubules (d). [file 13071_2025_7025_MOESM5_ESM.tif]
